# The Effect of Changing Fellowship Interview Format on Candidate Ranking Variabilities: The COVID-19 Experience

**DOI:** 10.1155/2022/7402079

**Published:** 2022-04-13

**Authors:** Ta Chen Chang, Elizabeth A. Hodapp, Richard K. Parrish, Alana L. Grajewski, Steven J. Gedde, Richard K. Lee, Sarah R. Wellik, Anna K. Junk, Luis Vazquez, Swarup S. Swaminathan, Elena Bitrian, Adam L. Rothman, Elizabeth A. Vanner

**Affiliations:** ^1^Bascom Palmer Eye Institute, University of Miami Miller School of Medicine, Miami, FL, USA; ^2^Miami Veterans Affairs Healthcare System, Miami, FL, USA

## Abstract

**Purpose:**

This study investigated the effect of interview format changes (in-person to virtual, one-to-one to multiple-to-one) necessitated by the COVID-19 travel restrictions on preliminary fellowship candidate ranking variabilities.

**Design:**

Cross-sectional observational study.

**Method:**

In 2018 and 2019, the glaucoma fellowship interviews were conducted in-person in a one-to-one format, whereas in 2020, interviews were virtual and in a multiple (interviewers)-to-one (candidate) format. We compared ranking ranges of interviewers within the same virtual room (WSR) and not within the same virtual room (NWSR) to assess the effect of WSR versus NWSR on ranking variabilities. We also compared ranking categories (“accept,” “alternate,” and “pass”) agreements between 2018, 2019, and 2020 to assess the effect of virtual versus in-person interviews on ranking variabilities.

**Results:**

NWSR and WSR mean rankings differed by 1.33 (95% confidence interval difference 0.61 to 2.04, *p* = 0.0003), with WSR interviewers having less variability than NWSR pairs. The variability between 2018/2019 (in-person interviews) and 2020 (virtual interviews) showed no differences between in-person and virtual interviews (weighted Kappa statistic 0.086 for 2018, 0.158 for 2019, and 0.101 for 2020; *p* < 0.05 for all years). The overall least attractive candidate has the lowest variability; the most attractive candidate has the second lowest variability.

**Conclusion:**

Grouping interviewers WSR during the interview decreased ranking variabilities compared to NWSR, while a change from in-person to virtual interview format did not increase the ranking variabilities. This suggests that the decreased nonverbal interactions in virtual interviews do not decrease interviewers' perceptions as applied to preliminary rankings.

## 1. Introduction

The fellowship interview is an important component of the selection process. For the applicants, the fellowship interview allows them to assess the training environment and culture, become acquainted with the program faculty members, and present themselves in the best light to future mentors. For the training program faculties, the interview allows assessment of the applicants' maturity and self-confidence, and their ability to articulate thoughts, listen well, and ask relevant questions [1]. The interview was found to be the single most important factor used in resident selection within the field of ophthalmology and other specialties of medicine [2, 3]. Similarly, prior surveys of program directors identified the interview and communication skills as criteria weighed most heavily in the selection of fellows in oculoplastics, retina, glaucoma, cornea, and oculoplastics [4, 5]. The interview was often the most important factor in ranking fellowship candidates in other medical specialties [6, 7]. In 2020, the coronavirus disease pandemic (COVID-19) and related travel restrictions made in-person interviews infeasible for most ophthalmology fellowship programs. As a result, most programs adopted virtual interviews via videoconferencing as well as other changes in format such as switching from the one-to-one interview format to multiple faculty-on-one interviews to economize the interview time periods. The impact of these changes on candidate ranking is not well understood.

In this study, we compared faculty interviewers' preliminary candidate rankings to identify the impact of the new interview formats on ranking variabilities. We hypothesized that in 2020, there may be significantly better agreement among interviewers who were assigned to the same virtual interview rooms compared to those that were in different rooms. Furthermore, the year 2020 agreement may be significantly better than the years 2018 and 2019 agreement due to fewer nonverbal assessments of the candidates as a result of virtual interview. To test these hypotheses, we performed a comparison of ranking variabilities between different interviewer-pairs for the 2020 interviews, and compare the candidate ranking categories between 2018, 2019, and 2020 interviews. A preprint has previously been published [8].

## 2. Methods

The study protocol has been reviewed and was determined to be exempt by the Institutional Review Board of the University of Miami Miller School of Medicine.

### 2.1. Overall Design

We analyzed the individual candidate rankings produced by the interviewers to elucidate the impact of a change from in-person, one-to-one interviews (years 2018 and 2019) to virtual, multiple-to-one interviews (year 2020). Specifically, we assessed the variabilities in candidate rankings in two ways: First, for the year 2020, we compared candidate ranking variabilities between interviewers who were within the same virtual interview room (“within the same room,” WSR) to those who were not within the same room (NWSR). Second, we compared candidate ranking variabilities between interviewers (who had interviewed all three years 2018–2020) to see if 2020 (virtual interviews) had less variabilities compared to prior years (in-person interviews), specifically avoided comparing interviewers who were WSR in 2020 to avoid WSR as a confounding factor.

### 2.2. Glaucoma Fellow Selection Process

Each year following the application deadline, applications for the Bascom Palmer Eye Institute clinical glaucoma fellowship were downloaded from the SF Match Residency and Fellowship Match Services (www.sfmatch.com) and screened by a committee of full-time glaucoma faculty members. From this application pool, a portion of qualified candidates were invited for an interview. In 2018 and 2019, the interviews were conducted in-person and one-to-one, whereas in 2020, the interviews were conducted virtually using a Zoom platform (Zoom Video Communications Inc., San Jose, CA) and two-on-one (faculty to applicant) or three-on-one. For each year, all interviews were completed in the same day. As the interview day progressed, each faculty interviewer was asked to individually rank the candidates from 1 (the most attractive candidate) to the highest number (the least attractive candidate). It was required that all ranking start with 1, be consecutive, and no two candidates may share the same rank. Following the interviews, these individual interviewers' preliminary candidate rankings were pooled, and a composite rank list was generated, with the candidate with the rank closest to 1 being the most attractive, and the one farthest from 1 being the least attractive. The committee then adjusted the rankings in a structured discussion. More than one discussion meeting may have occurred before a finalized rank list is submitted to SF Match.

### 2.3. Interview Formats

There were no overt instructions to the interviewers or structures imposed on the individual interviews except to remain within the allowed time slot in order to stay on schedule. In 2018 and 2019, the candidates were invited on-site at the Miami campus of the Bascom Palmer Eye Institute (Miami, FL), and the interviews were conducted in-person in a one-to-one format. The interviews took place in individual faculty members' offices. A facilitator was used to keep time and to direct candidates from one interview room to the next. In 2020, the interviews were conducted via the Internet using a virtual online meeting format. Rather than one-to-one, there were five virtual rooms each with two or three faculty members. In all years, each interview was allotted a 15-minute time slot. Two warnings were given when there were three- and one-minute remaining. In all years, all candidates had the opportunity to interview with all participating faculty interviewers. In the 2020 virtual interviews, no two interviewers occupied the same physical space. The interviewers' candidate rankings were created individually, prior to any group discussion of candidate qualifications.

### 2.4. Internal Candidates

Since the goal of the study was to assess the effect of different interview settings and formats on candidate ranking variabilities, internal candidates (Bascom Palmer Eye Institute residents and/or research fellows applying for clinical glaucoma fellowships) were removed from the database and excluded from analysis, as the interviewers had frequent interactions with them outside the glaucoma fellowship application process. Once the internal candidates were removed from the data sets, each interviewer's rankings were adjusted by collapsing the removed rank. For example, if an interviewer had ranked the internal candidate as 3, then, all subsequent rankings were reduced by 1 (so the person ranked 4 was adjusted to 3, the person ranked 5 was adjusted to 4, and so on). This adjustment was made to all candidate rank orders for all three years.

### 2.5. Defining Variability and Outcome Categories

For ordinal ranked data, the range is the most appropriate statistic to assess variability within the same year. The range was determined for each candidate for each pair of interviewers. For 2020, each range was designed as WSR if that pair of interviewers were in the same virtual interview room and NWSR otherwise. To compare 2018, 2019, and 2020, we included pairs of interviewers only among those faculty members who interviewed during all 3 years. Each included faculty member was paired with all other included faculty members (except those with which he/she was WSR in 2020), and the ranges were determined for each year. However, since different numbers of candidates were interviewed each year, this resulted in different rank ranges for each year, and analyses across different years based on the range of ranks could be confounded. To avoid this problem, when comparing across different years, we translated the rankings into 3 categories: accept, alternate, and pass. Each year, four glaucoma fellows are appointed, so each interviewer's top 4 candidates were categorized as accept, the next 4 candidates were categorized as alternate, and all other candidates were categorized as pass. This categorization approach translated different rank ranges into the same 3 categories for the 3 years and thus avoids confounding factors due to different rank ranges between the 3 interview years.

### 2.6. Statistical Methods

Categorical and ranked variables are presented with frequencies and percentages. We assessed the variability of the ranges (of the ranks for pairs of interviewers) between 2018, 2019 (in-person interviews) and 2020 (virtual interviews) using a general linear model (GLM). Similarly, for 2020 interviews, we assessed the variability of the ranges of WSR interviewer pairs to NWSR pairs using a GLM. We used weighted Kappa statistics to compare the agreement of interviewer rankings placed into the 3 categories: accept, alternate, and pass for 2018, 2019, and 2020. Similarly, we used weighted Kappa statistics to compare the agreement of interviewer rankings placed into the 3 categories: accept, alternate, and pass between the WSR pairs and the NWSR pairs for the 2020 interviews. In all interyear comparisons, interviewer-pairs that shared a virtual interview room in 2020 were omitted to avoid the effect of sharing a virtual interview room on individual variabilities, since there had been no shared rooms in 2018 and 2019. All analyses were performed using the SAS version 9.4 software (SAS Institute, Cary, NC, USA). A *p* value ≤0.05 was considered statistically significant, and a *p* value between 0.05 and 0.15 was considered marginally significant. SAS does not produce precise *p* values for weighted (ordinal) Kappa statistics, nor for comparisons between two weighted Kappa statistics.

## 3. Results

For 2018 and 2019, rankings were from 1 to 20 (21 candidates including one internal candidate interviewed each of the two years), and for 2020, rankings were from 1 to 18 (19 candidates including one internal candidate interviewed). There were 8, 9, and 11 interviewers in 2018, 2019, and 2020, respectively, 7 of which interviewed in all 3 years.

Effect of interviewers being within the same virtual rooms versus different virtual rooms on ranking variability.

There were 11 interviewers in 2020, which were separated into 5 different virtual interview rooms (4 rooms with 2 interviewers, 1 room with 3) and results in the permutation of 7 WSR pairs (the 3-interviewer room has a permutation of 3 pairs) and 48 NWSR pairs. The ranking range spanned between 0 (no difference in ranking range) to 17 (widest possible range), with the most common ranking difference being “2” amongst all WSR (*n* = 28, 22.2%) and NWSR (*n* = 114, 13.2%) pairs. Overall, the mean difference in the ranks between NWSR and WSR was 1.33 (95% confidence interval (CI) difference 0.61 to 2.04, *p* = 0.0003), with the NWSR pairs having a greater variability (greater difference in the ranks of the 2 interviewers) than the WSR pairs. This implies that WSR interviewers, on average, ranked candidates 1.33 places closer to each other than NWSR interviewers. Furthermore, when comparing the rank range of each of the 7 WSR pairs to the 48 NWSR pairs, WSR pairs ranked candidates closer to each other than NSWR pairs, although this difference is only significant or marginally significant in 3 of the 7 comparisons (Table 1).

**Figure 1:**
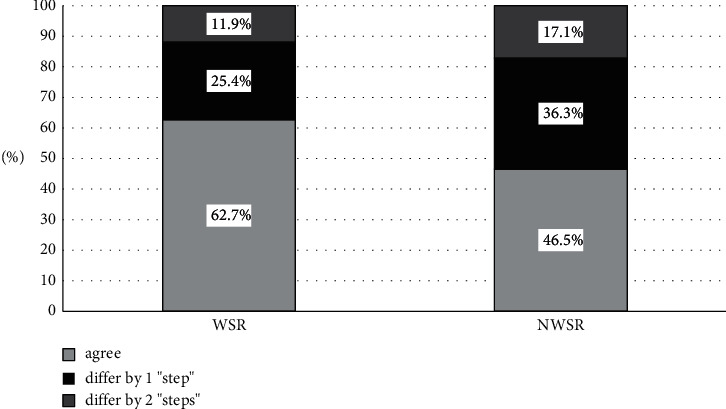
Candidate category (“accept,” “alternate,” and “pass”) agreements between pairs of interviewers who were within the same virtual interview room (WSR) and not within the same virtual interview room (NWSR); “differ by 1 step” denotes a difference between “accept” and “alternate” or “alternate” and “pass,” whereas “differ by 2 steps” denotes a difference between “accept” and “pass.”

When evaluating the variability of candidate categories, WSR pairs agreed 53.6%, 42.9%, and 74.3% on “accept,” “alternate,” and “pass” (weighted Kappa 0.41, 95% CI Kappa 0.27 to 0.56, *p* < 0.050) (Table 2), whereas NWSR had significantly less agreement at 30.7%, 18.2%, and 64.2% for the same 3 categories (weighted Kappa 0.16, 95% CI Kappa 0.10 to 0.21, *p* < 0.05). The weighted Kappa of NWSR is below the lower limited of the 95% CI Kappa of WSR, suggesting that there is significantly greater degree of agreement in the categorization of candidates for the WSR interviewers than for the NWSR interviewers (Figure 1).

### 3.1. Effect of In-Person versus Virtual Interviews on Ranking Variability

A 3-way comparison of variability between 2018/2019 (in-person interviews) and 2020 (virtual interviews) ranking variabilities amongst interviewers who were present at all three years showed no significant differences between in-person and virtual interviews (Table 3). When comparing agreement of candidates being in “accept,” “alternate,” and “pass” categories, the weighted Kappa statistic was 0.086 for 2018, 0.158 for 2019, and 0.101 for 2020 (*p* < 0.05 for all years). Since the weighted Kappa statistic for 2020 is within the 95% CI of the weighted Kappa statistic for both 2018 and 2019, the 2020 virtual interviews did not result in a greater degree of disagreement in the categorization of candidates when compared to the in-person interviews in 2018 and/or 2019 (Table 4).

### 3.2. Candidates with the Least and Greatest Ranking Variabilities

For 2020, we assessed the variability of rank ranges for the overall most and the least attractive candidates. The overall least attractive candidate has the lowest variability (indicating a high degree of agreement amongst the interviewers), while the most attractive candidate has the second lowest variability. The greatest discordances were with the candidate ranked 8th (GLM estimated average difference = 5.93) and the candidate ranked 5th (GLM estimated average difference = 5.31). However, a candidate's overall composite ranking (1 to 18) was not significantly associated with the variability in the individual pairs' rankings (*p* = 0.8597).

## 4. Discussion

The COVID-19 pandemic has significantly altered many processes in academic medicine, such as the oral examination for board certification as well as the residency and fellowship interviews for most training programs. In our institution, a change from one-to-one interviews to multiple-to-one produced less variabilities in candidate rankings amongst interviewers who shared the same virtual room. This is likely due to decreased variety of questions from the interviewers, although cognitive biases due to groupthink and herd effects may allow the more influential member of the interview team to overshadow dissenting opinions. [9] If the program's goal were to allow the candidates to represent themselves in the best light, then, fewer group interviews may be less desirable compared to numerous individual, one-to-one interviews.

The virtual interviews in 2020 resulted in similar ranking variabilities compared to in-person interviews from 2018 to 2019 among interviewers who were not in the same virtual interview room in 2020. While the limits of videoconferencing on nonverbal body language perception may present a barrier to adequate care in telemedicine, [10, 11] it does not seem to affect candidate rankings when compared to in-person interviews. This is supported by two prior surveys in which candidates to surgical fellowships felt that they were able to present themselves satisfactorily to the training program through a videoconferencing format when compared to in-person interviews. [12, 13] In our clinical training program, the candidates with the least and second least variabilities were the least and most desired candidates, suggesting that individual opinions converge at the most and least qualified applicants. However, the candidates with the most variabilities (thus with the most disagreement) were ranked 5^th^ and 8^th^, which are on the cusps of being from the “alternate” to either the “accepted” or “pass” categories in our program which matches four clinical fellows per year, and perhaps the interview committee's greatest deliberation effort would be spent on adjudicating these two positions.

There are several limitations to our study. First, we do not know whether the finding of decreased ranking variabilities of WSR interviewers would have occurred if they were in the same physical rooms. Second, our results relate to fellowship selection in one subspecialty at a single academic institution, which may limit their generalizability. Third, we could not gather data on the candidates' ranking variabilities of glaucoma fellowship programs to assess whether the effects we have observed applies to the candidates. Despite the lack of differences in variabilities in virtual interviews compared to in-person interviews, prior studies showed that most candidates consider the in-person interview experience to be better than virtual interview experience, and in-person interview candidates felt better acquainted with the faculty and current trainees compared to virtual interview candidates [14]. Furthermore, in-person interview candidates were also more likely to agree that the interview experience was sufficient to allow them to make a ranking decision [15], while many virtual interview candidates felt that they did not get an adequate understanding of the program [16].

In summary, in our clinical glaucoma fellowship program, a change from one-to-one to multiple-to-one interview resulted in less candidate ranking variabilities between interviewers paired in the rooms, which may decrease the candidates' opportunities to represent themselves well. A change from in-person to virtual interviews did not result in greater candidate ranking variability, while the greatest agreements occurred with the least and most attractive candidates. As ophthalmology training programs enter the post-COVID-19 era, these findings will help to restructure the trainee selection process and to strike a balance between maximizing the opportunities for programs and candidate to learn about each other while decreasing travel burden.

## Figures and Tables

**Table 1:** Comparison of ranking variabilities of interviewers who shared the virtual interview rooms to those who did not share a virtual interview room.

Groups compared	Mean				
	Diff.	95% CI diff.	
	(Beta)	Low	High	*p* value	
NWSR to WSR ALL	1.33	0.61	2.04	0.0003	^*∗∗∗*^
NWSR to WSR 1	−3.03	−4.81	−1.24	0.0009	^*∗∗∗*^
NWSR to WSR 2	−1.03	−2.81	0.76	0.2592	
NWSR to WSR 3	−1.47	−3.26	0.31	0.1062	†
NWSR to WSR 4	−0.92	−2.70	0.87	0.3143	
NWSR to WSR 5	−1.14	−2.93	0.65	0.2113	
NWSR to WSR 6	−0.36	−2.15	1.43	0.6917	
NWSR to WSR 7	−1.36	−3.15	0.43	0.1352	†

NWSR, not within the same room; WSR, within the same room.^*∗*^*p* < 0.05, ^*∗∗*^*p* < 0.01, ^*∗∗∗*^*p* < 0.001, †*p* < 0.15 (marginally significant). Mean diff. (beta), mean difference resulted from WSR subtracting NWSR rank range; a negative value denotes greater variability within the NWSR pairs when compared to WSR pairs.

**Table 2:** Agreement of candidate categories between pairs of interviewers that are within the same room and not within in the same room

Ranking Categories	Interviewer #1 (below)	Interviewer #2			Weighted	95% CI Kappa		Kappa*P*<0.05	Is Kappa in CI of other Kappa?
		Accept	Alternate	Pass	Kappa	Low	High		NWSR	
Interviews within the same room	Accept	15 (53.6%)	5 (17.9%)	8 (11.4%)	0.414	0.271	0.557	Yes	No	
	Alternate	6 (21.4%)	12 (42.9%)	10 (14.3%)						
	Pass	7 (25%)	11 (39.3%)	52 (74.3%)						
Interviews not within the same room	Accept	59 (30.7%)	62 (32.3%)	71 (14.8%)	0.159	0.104	0.214	Yes		
	Alternate	56 (29.2%)	35 (18.2%)	101 (21%)						
	Pass	77 (40.1%)	95 (49.5%)	308 (64.2%)						

CI, confidence interval; NSWR, not within the same room; WSR, within the same room. Weighted Kappa outside CI of the other Kappa means there was significant differences in candidate category agreement between WSR and NWS

**Table 3:** Comparison of ranking variabilities of in-person interviews (2018 and 2019) with virtual interviews (2020).

Groups compared	Mean			
	Diff.	95% CI diff.	
	(Beta)	Low	High	*p* value
2018 to 2019	−0.174	−0.780	0.433	0.5743
2018 to 2020	0.386	−0.237	1.009	0.2245
2019 to 2020	0.560	−0.063	1.183	0.0783

^*∗*^ *p* < 0.05, ^*∗∗*^*p* < 0.01, ^*∗∗∗*^*p* < 0.001, ^†^*p* < 0.15 (marginally significant). Mean diff. (beta), mean difference resulted from subtracting the rank range from one year to another.

**Table 4:** Agreement of candidate categories between pairs of interviewers between in-person interviews (2018 and 2019) and virtual interviews (2020).

Ranking Categories	Interviewer #1 (below)	Interviewer #2			Weighted	95% CI Kappa		Kappa	Is Kappa in CI of other Kappa?	
		Accept	Alternate	Pass	Kappa	Low	High	*P*<0.05	2019	2018
2018	Accept	17 (22.4%)	18 (23.7%)	41 (18%)	0.086	0.002	0.169	Yes		
	Alternate	23 (30.3%)	14 (18.4%)	39 (17.1%)						
	Pass	36 (47.4%)	44 (57.9%)	148 (64.9%)						

2019	Accept	24 (31.6%)	17 (22.4%)	35 (15.4%)	0.158	0.072	0.244	Yes		
	Alternate	20 (26.3%)	15 (19.7%)	41 (18%)						
	Pass	32 (42.1%)	44 (57.9%)	152 (66.7%)						

2020	Accept	20 (26.3%)	25 (32.9%)	31 (16.3%)	0.101	0.015	0.188	Yes	Yes^*∗*^	Yes^*∗*^
	Alternate	21 (27.6%)	13 (17.1%)	42 (22.1%)						
	Pass	35 (46.1%)	38 (50%)	117 (61.6%)						

CI, confidence interval. ^∗^Weighted Kappa within CI of other Kappa means there are no significant differences in candidate category agreement between 2020 and the other years.

## Data Availability

The data used to support the findings of this study are available on request.
